# Pyrogallic Acid in Soft Chancre

**Published:** 1882-01

**Authors:** 


### PYROGALLIC ACID IN SOFT CHANCRE.

*Le Siecle Medical* gives the following formula as used by M. Terrillon and others: Starch 10.0, vaseline 30.0 pyrogallic acid 10.0. The ointment should be preserved in a glass-stoppered bottle. The pain from the application is not severe and does not often last over five minutes. The application to the anus causes more pain than at the vulva. If, however, a bubo be opened and afterwards takes on a chancroid form the application of the ointment causes severe pains; more painful when the edges of the incision assume a chancroid condition than when the wound is markedly phagedenic. Cicatrization is rapid. After the application the sore becomes cleaner and has a light whitish tint, due to the slight caustic action. At times if the application is made more frequently than twice a week, a brownish pellicle appears which afterwards forms a black crust. This occurs more especially in the phagedenic chancres and is a favorable prognostic. The falling of the crust leaves a red and granulating wound behind, which soon cicatrizes.—*N. Y. Med. Times.*

